# Effect of stacking insecticidal *cry* and herbicide tolerance *epsps* transgenes on transgenic maize proteome

**DOI:** 10.1186/s12870-014-0346-8

**Published:** 2014-12-10

**Authors:** Sarah Zanon Agapito-Tenfen, Vinicius Vilperte, Rafael Fonseca Benevenuto, Carina Macagnan Rover, Terje Ingemar Traavik, Rubens Onofre Nodari

**Affiliations:** CropScience Department, Federal University of Santa Catarina, Rod. Admar Gonzaga 1346, 88034-000 Florianópolis, Brazil; Genøk Center for Biosafety, The Science Park, P.O. Box 6418, 9294 Tromsø, Norway

**Keywords:** Genetically modified organisms, Stacked GMO, Pyramiding, Bt Crops, Molecular profiling, Risk assessment, Glyphosate

## Abstract

**Background:**

The safe use of stacked transgenic crops in agriculture requires their environmental and health risk assessment, through which unintended adverse effects are examined prior to their release in the environment. Molecular profiling techniques can be considered useful tools to address emerging biosafety gaps. Here we report the first results of a proteomic profiling coupled to transgene transcript expression analysis of a stacked commercial maize hybrid containing insecticidal and herbicide tolerant traits in comparison to the single event hybrids in the same genetic background.

**Results:**

Our results show that stacked genetically modified (GM) genotypes were clustered together and distant from other genotypes analyzed by PCA. Twenty-two proteins were shown to be differentially modulated in stacked and single GM events versus non-GM isogenic maize and a landrace variety with Brazilian genetic background. Enrichment analysis of these proteins provided insight into two major metabolic pathway alterations: energy/carbohydrate and detoxification metabolism. Furthermore, stacked transgene transcript levels had a significant reduction of about 34% when compared to single event hybrid varieties.

**Conclusions:**

Stacking two transgenic inserts into the genome of one GM maize hybrid variety may impact the overall expression of endogenous genes. Observed protein changes differ significantly from those of single event lines and a conventional counterpart. Some of the protein modulation did not fall within the range of the natural variability for the landrace used in this study. Higher expression levels of proteins related to the energy/carbohydrate metabolism suggest that the energetic homeostasis in stacked versus single event hybrid varieties also differ. Upcoming global databases on outputs from “omics” analyses could provide a highly desirable benchmark for the safety assessment of stacked transgenic crop events. Accordingly, further studies should be conducted in order to address the biological relevance and implications of such changes.

**Electronic supplementary material:**

The online version of this article (doi:10.1186/s12870-014-0346-8) contains supplementary material, which is available to authorized users.

## Background

The first decade of GM crop production has been dominated by genetically modified (GM) plants containing herbicide tolerance traits, mainly based on Roundup Ready® herbicide (Monsanto Company) spray, and on insect protection conferred by Cry proteins-related traits, also called ‘Bt toxins’. More recently, GM crop cultivation has been following a trend of products combining both traits by traditional breeding. In the existing literature, such combinations are referred to as “stacked” or “pyramided” traits or events [1]. In recent years, an increasing number of GM plants that combine two or more transgenic traits reached about 47 million hectares equivalent to 27% of the 175 million hectares planted with transgenic crops worldwide in 2013, up from 43.7 million hectares or 26% of the 170 million hectares in 2012 [2].

According to the current regulatory practice within the European Union (EU), stacked events are considered as new GM organisms: prior to marketing they need regulatory approval, including an assessment of their safety, similar to single events [3]. In other countries, like Brazil, stacked events are also considered new GMOs but do not require full risk assessments if single parental events have been already approved. In other words, there is a simplified risk assessment procedure (provided by Normative Resolution n^o^ 8/2009) that requires less safety studies than those under first time approval [4]. In the United States, for example, this is not even obligatory [5].

To comply with current international guidance on risk assessment of stacked GM events, additional information on the stability of transgene insertions, expression levels and potential antagonistic or synergistic interactions on transgenic proteins should be provided [6,7].

Literature on molecular characterization of GM stacked events is scarce, and the comparison of their expression levels and potential cellular interaction to parental single GM lines is absent. Few recent studies about the possible ecological effects of stacked GM crops have been published, but frequently lack the comparison to the GM single lines or even the near-isogenic non-transgenic line [8-10]. In addition, the approach taken by these authors was to assess potential adverse effects of stacked transgenic crop products such as pollen and grain. This approach does not isolate the unique effects of stacking two or more transgenic inserts. Neither has it identified intended and unintended differences nor equivalences between the GM plant and its comparator(s). Earlier published literature also failed to recognize potential interactions between the events present or their stability. GM plants containing stacked events cannot be considered generally recognized as safe without specific supporting evidence [3].

Profiling techniques, such as proteomics, allow the simultaneous measurement and comparison of thousands of plant components without prior knowledge of their identity [11]. The combination of target and non-target methods allows a more comprehensive approach, and thus additional opportunities to identify unintended effects of the genetic modification are provided [12].

Accordingly, our novel approach uses proteomics as a molecular profiling technique to identify potential unintended effects resulting from the interbreeding of GM varieties (e.g. synergistic or antagonistic interactions of the transgenic proteins). The aim of this study was to evaluate protein changes in stacked versus single event and control plants under highly controlled conditions, to examine the expression levels of transgenic transcripts under different transgene dosage (one or two transgene insertions) and to provide insight into the formulation of specific guidelines for the risk assessment of stacked events. We hypothesized that the combination of two transgenes could differentially modulate endogenous protein expression, which might have an effect on the plant metabolism and physiology. In addition, the expression levels of two transgenes may be altered in GM stacked events relative to single transformation events. To test these hypotheses, we have used GM stacked maize genotype containing *cry1A.105/cry2Ab2* and *epsps* cassettes expressing both insect resistance and herbicide tolerance as unlinked traits, as well as genotypes of each single transgene alone, being all maize hybrids in the same genetic background. The seed set of stacked and single GM maize events, as well as the conventional near-isogenic counterpart developed in the same genetic background and a landrace variety, enables the isolation of potential effects derived from stacking two transgenes. Finally, we have performed two dimensional differential gel electrophoresis analysis (2D-DIGE) and quantitative Real-Time PCR experiments (RT-qPCR) to determine differences in the proteome and transcription levels of transgenes between stacked and single events.

## Methods

### Plant material and growth chamber conditions

Five maize varieties were used in this study. Two of them are non-GM maize seeds, the hybrid AG8025 (named here as ‘conventional’) from Sementes Agroceres and the open pollinated variety Pixurum 5 (named here as ‘landrace’). Pixurum 5 has been developed and maintained by small farmers in South Brazil for around 16 years [13].

The other three varieties are GM and have the same genetic background as the conventional variety since they are produced from the same endogamic parental lines. These are: AG8025RR2 (unique identifier MON-ØØ6Ø3-6 from Monsanto Company, glyphosate herbicide tolerance, Sementes Agroceres); AG8025PRO (unique identifier MON-89Ø34-3 from Monsanto Company, resistance to lepidopteran species, Sementes Agroceres) and AG8025PRO2 (unique identifier MON-89Ø34-3 × MON-ØØ6Ø3-6 from Monsanto Company, stacked event resistant to lepidopteran species and glyphosate-based herbicides, Sementes Agroceres). These are named in this study as RR, Bt and RRxBt, respectively (Table 1). The AG8025 variety is the hybrid progeny of the single-cross between maternal endogamous line “A” with the paternal endogamous line “B”. Thus, the used hybrid variety seeds have high genetic similarity (most seeds should be AB genotype). All these five commercial varieties were produced by the aforementioned company/farmers and are commonly found in the market in Brazil.

**Table 1:** **Transgenic and non-transgenic comercial maize hybrid varieties used in this study**

**Maize variety comercial name**	**GM event**	**Transgenes**	**N** ^**o**^ **of samples (individual plants)**	**Designated in this study**
AG8025PRO2	MON-89Ø34-3 x MON-ØØ6Ø3-6	cry1A.105/cry2Ab2 x epsps/epsps	15	RRxBt samples
AG8025PRO	MON-89Ø34-3	cry1A.105/cry2Ab2	15	Bt samples
AG8025RR2	MON-ØØ6Ø3-6	epsps/epsps	15	RR samples
AG8025	n.a	n.a.	15	Conventional samples
Pixurum 5	n.a.	n.a.	15	Landrace samples

Transgenic maize varieties and its corresponding transformation events, plus containing transgenes, were described in the following rows. The numbers of individual plants sampled per maize variety, as well as their designation, are also provided.

Note: Not applied (n.a.).

The cultivation of MON-ØØ6Ø3-6, MON-89Ø34-3, and MON-89Ø34-3 × MON-ØØ6Ø3-6 has been approved in Brazil in 2008 [14], 2009 [15] and 2010 [16] respectively. The stacked hybrid MON-89Ø34-3 × MON-ØØ6Ø3-6 expresses two insecticidal proteins (Cry1A.105 and Cry2Ab2 proteins derived from *Bacillus thuringiensis*, which are active against certain lepidopteran insect species) and two identical EPSPS proteins providing tolerance to the herbicide glyphosate. The novel traits of each parent line have been combined through traditional plant breeding to produce this new hybrid. The experimental approach currently applied for the comparative assessment requires the use of conventional counterpart and the single-event counterparts, all with genetic background as close as possible to the GM plant, as control [6,7,17].

After the confirmation by PCR of the transgenic events in both single and stacked GM seeds and the absence in the conventional and landrace ones (data not shown), the seeds from all the five varieties were grown side by side in growth chambers (Eletrolab^TM^ model 202/3) set to 16 h light period and 25 °C (± 2 °C). Seedlings were germinated and grown in Plantmax HT substrate (Buschle & Lepper S.A.) and watered daily. No pesticide or fertilizer was applied. Around 50 plants were grown in climate chambers out of which fifteen plants were randomly sampled per maize variety (genotype). The collected samples were separated in three groups of five plants. The five plants of each group were pooled and were considered one biological replicate. Maize leaves were collected at V4 stage (20 days after seedling). Leaf pieces were cut out, weighed and placed in 3.8 ml cryogenic tubes before immersion in liquid nitrogen. The samples were kept at −80 °C until RNA and protein extraction. This experiment was repeated and a second relative quantification analysis of transgene transcripts was performed in order to reproduce the results.

### RNA isolation and relative quantification analysis of transgene transcripts

RNA was extracted from approximately 100 mg of frozen leaf tissue using RNeasy Plant Mini Kit (Qiagen, Hilden, Germany) according to the manufacturer’s instructions. In brief, samples were homogenized with guanidine-isothiocyanate lysis buffer and further purified using silica-membrane. During purification, in-column DNA digestion was performed using RNAse-free DNAse I supplied by Qiagen to eliminate any remaining DNA prior to reverse transcription and real-time PCR. The extracted RNA was quantified using NanoDrop 1000 (Thermo Fisher Scientific, Wilmington, USA).

Reverse-transcription quantitative PCR (RT-qPCR) assay was adapted from previously developed assays for the specific detection of MON-89Ø34-3 × MON-ØØ6Ø3-6 transgenes [18] to hydrolysis ZEN - Iowa Black® Fluorescent Quencher (ZEN/ IBFQ) probe chemistry (Integrated DNA Technologies, INC Iowa, USA).

Following quantification, cDNA was synthesized and amplification of each target gene was performed using the QuantiTect Probe RT-PCR Kit (Qiagen) according to the manufacturer’s instructions. RT-qPCR experiment was carried out in triplicates using StepOne™ Real-Time PCR System (Applied Biosystems, Singapore, Singapore). Each 20 μl reaction volume comprised 10 uM of each primer and probe and 50 ng of total RNA from each sample. The amplification efficiency was obtained from relative standard curves provided for each primer and calculated according to Pfaffl equations [19].

The two most suitable endogenous reference genes out of five candidates (ubiquitin carrier protein, folylpolyglutamate synthase, leunig, cullin, and membrane protein PB1A10.07c) were selected as internal standards. The candidate genes were chosen based on the previous work of Manoli et al. [20]. The selection of the two best endogenous reference genes for this study was performed using NormFinder (Molecular Diagnostic Laboratory, Aarhus University Hospital Skejby, Denmark) statistical algorithms [21]. Multiple algorithms have been devised to process RT-qPCR quantification cycle (Cq). However, NormFinder algorithm has the capability to estimate both intragroup and intergroup variance and the identification of the two reference genes as most stable normalizers [22]. The leunig and membrane protein PB1A10.07c genes were used to normalize *epsps*, *cry1a.105* and *cry2ab2* mRNA data due to their best stability value (SV for best combination of two genes 0.025, data not shown). Conventional samples were also analyzed in order to check for PCR and/or seed contaminants. Primer and probe sequences used, as well as Genebank ID of target genes, are provided in Additional file 1. The primers and probes were assessed for their specificity with respect to known splice variants and single-nucleotide polymorphism positions documented in transcript and single-nucleotide polymorphism databases.

The normalized relative quantity (NRQ) was calculated for stacked transgenic event samples relative to one of the three-pooled samples correspondent to the single transgenic event according to the Pfaffl equations [19].

### Protein extraction and fluorescence hybridization

Approximately 100 mg of each sample was separately ground-up in a mortar with liquid nitrogen, and protein extraction was subsequently carried out according to Carpentier et al. [23], with some modification. Phenol extraction and subsequent methanol/ammonium acetate precipitation were performed and PMSF was used as protease inhibitor. Pellets were re-suspended in an urea/thiourea buffer compatible to further fluorescent labeling (4% w/v CHAPS, 5 mM PMSF, 7 M urea, 2 M thiourea and 30 mM Tris-base). Protein quantification was determined by means of a copper-based method using 2-D Quant Kit (GE Healthcare Bio-Sciences AB, Uppsala, Sweden). Before sample storage in −80 °C, 80 ug of each protein sample pool were labeled with 400 ρmol/ul of CyDye DIGE fluors (Cy3 and Cy5; GE Healthcare), according to the manufacturer’s instructions. An internal standard for normalization was used in every run; this was labeled with Cy2. The internal standard is a mixture of equal amounts of each plant variety sample. After protein-fluor hybridization, samples were treated with lysine (10 mM) to stop the reaction and then mixed together for 2D-DIGE gel electrophoresis separation. Sample pairs were randomly selected for two-dimensional electrophoresis runs.

### 2D-DIGE gel electrophoresis conditions

After protein labeling, samples were prepared for isoelectric focusing (IEF) step. Strip gels of 24 cm with a linear pH range of 4–7 (GE Healthcare) were used. Strips were initially rehydrated with labeled protein samples (7 M urea, 2 M thiourea, 2% w/v CHAPS, 0.5% v/v IPG buffer (GE Healthcare), 2% DTT). Strips were then processed using an Ettan IPGPhor IEF system (GE Healthcare) in a total of 35000 Volts.h^−1^ and, subsequently, reduced and alkylated for 30 min under slow agitation in Tris–HCl solution (75 mM) pH 8.8, containing 2% w/v SDS, 29.3% v/v glycerol, 6 M urea, 1% w/v DTT and 2.5% w/v iodocetamide. Strips were placed on top of SDS-PAGE gels (12%, homogeneous) and used in the second dimension run with a Hoefer DALT system (GE Healthcare). 2D gel electrophoresis conditions were performed as described by Weiss and Görg [24]. Gels were immediately scanned with the FLA-9000 modular image scanner (Fujifilm Lifescience, Dusseldorf, Germany). To ensure maximum pixel intensity between 60 000 and 90 000 pixels for the three dyes, all gels were scanned at a 100 μm resolution and the photo multiplier tube (PMT) voltage was set between 500 and 700 V.

Preparative gels for each plant variety were also performed in order to extract relevant spots. These were performed with a 450 ug load of total protein pools in 24 cm gels from each variety, separately, and stained with coomassie brilliant blue G-250 colloidal (MS/MS compatible) as described by Agapito-Tenfen et al. [25].

### Image analysis

The scanned gel images were transferred to the ImageQuant V8.1 software package (GE Healthcare) for multiplexing colored DIGE images. After cropping, the images were exported to the software ImageMaster^TM^ 2D Platinum 7.0, version 7.06 (GE Healthcare) for cross comparisons between gels. Automatic spots co-detection of each gel was performed followed by normalization with the corresponding internal standard and matching of biological replicates and varieties. Manual verification of matching spots was applied. This process results in highly accurate volume ratio calculations. Landmarks and other annotations were applied for determination of spot experimental mass and pI (isoelectric point).

### In-gel digestion and protein identification by MS/MS

Spots from preparative gels were excised and sent to the Proteomic Platform Laboratory at the University of Tromsø, Norway, for processing and analysis. These were subjected to in-gel reduction, alkylation, and tryptic digestion using 2–10 ng/μl trypsin (V511A; Promega) [26]. Peptide mixtures containing 0.5% formic acid were loaded onto a nano ACQUITY Ultra Performance LC System (Waters Massachusetts, USA), containing a 5-μm Symmetry C18 Trap column (180 μm × 20 mm; Waters) in front of a 1.7-μm BEH130 C18 analytical column (100 μm × 100 mm; Waters). Peptides were separated with a gradient of 5–95% acetonitrile, 0.1% formic acid, with a flow of 0.4 μl/min eluted to a Q-TOF Ultima mass spectrometer (Micromass; Waters). The samples were run in data-dependent tandem MS mode. Peak lists were generated from MS/MS by the Protein Lynx Global server software (version 2.2; Waters). The resulting ‘pkl’ files were searched against the NCBInr 20140323 protein sequence databases using Mascot MS/MS ion search (Matrix Sciences; http://matrixscience.com). The taxonomy used was Viridiplantae (Green Plants) and ‘all entries’ and ‘contaminants’ for contamination verification. The following parameters were adopted for database searches: complete carbamidomethylation of cysteines and partial oxidation of methionines; peptide mass tolerance ± 100 ppm; fragment mass tolerance ± 0.1 Da; missed cleavages 1; and significance threshold level (*P* < 0.05) for Mascot scores (−10 Log (*P*)). Even though high Mascot scores are obtained with significant values, a combination of automated database searches and manual interpretation of peptide fragmentation spectra were used to validate protein assignments. Molecular functions and cellular components of proteins were searched against ExPASy Bioinformatics Resource Portal (Swiss Institute for Bioinformatics; http://expasy.org) and Kyoto Encyclopedia of Genes and Genomes (KEGG) Orthology system database release 69.0 2014 (http://kegg.jp/kegg/ko.html). In order to understand and interpret these data and to test our hypothesis on the systemic response of the proteomes we have generated, we have further classified and filtered the list of identified proteins for pathway abundances. The enrichment analysis to compare the abundance of specific functional biological processes has been performed using BioCyc Knowledge Library (http://biocyc.org/) [27] and their corresponding statistical algorithms. The proteins were searched against the maize (*Zea mays*) database.

### Statistical analysis

Real-time relative quantification data were plotted and manually analyzed using Microsoft Excel (Microsoft, Redmond, WA). Normalized gene expression data was obtained using the Pfaffl method for efficiency correction [19]. Cq average from each technical replicate was calculated for each biological replicate and used to make a statistical comparison of the genotypes/treatment based on the standard deviation. Due to non-normal distribution, the fold change data were log10 transformed. The fold change means obtained for single versus stacked GM event were compared using T-test at *P* <0.05 (R program software) [28]. Information on real-time data for this study has followed guidelines from the Minimum Information for Publication of Quantitative Real-Time PCR Experiments [29].

The main sources of variation in the 2D-DIGE experiment dataset were evaluated by unsupervised multivariate PCA, using Euclidean distance for quantitative analysis. PCA analyses were performed by examining the correlation similarities between the observed measures. The spot volume ratio was analyzed using covariance matrix on Multibase Excel Add-in software version 2013 (Numerical Dynamics; http://www.numericaldynamics.com). For the 2D-DIGE experiment, one-way ANOVA was used to investigate differences at individual protein levels. Tukey test at *P* < 0.05 was used to compare the multiple means in the dataset using R program software [28]. The calculations were performed on normalized spot volume ratios based on the total intensity of valid spots in a single gel. Differences at the level *P* < 0.05 were considered statistically significant. Statistical analyses were performed using ImageMaster^TM^ 2D Platinum 7.0, version 7.06 (GE Healthcare).

## Results and discussion

To examine potential unintended effects of combining transgenes by conventional breeding techniques, the protein expression profile, as well as transgenic mRNA levels, of stacked GM maize leaves expressing insecticidal and herbicide tolerance characteristics were evaluated in comparison to four other maize genotypes. These were two single event GM hybrids with the same genetic background; the conventional counterpart non-GM hybrid AG8025 and a landrace variety (Pixurum 5) exposed to highly controlled growth conditions.

### Transcript levels of *epsps*, *cry1A.105* and *cry2Ab2* in leaves of stacked GM maize

**Figure 1:**
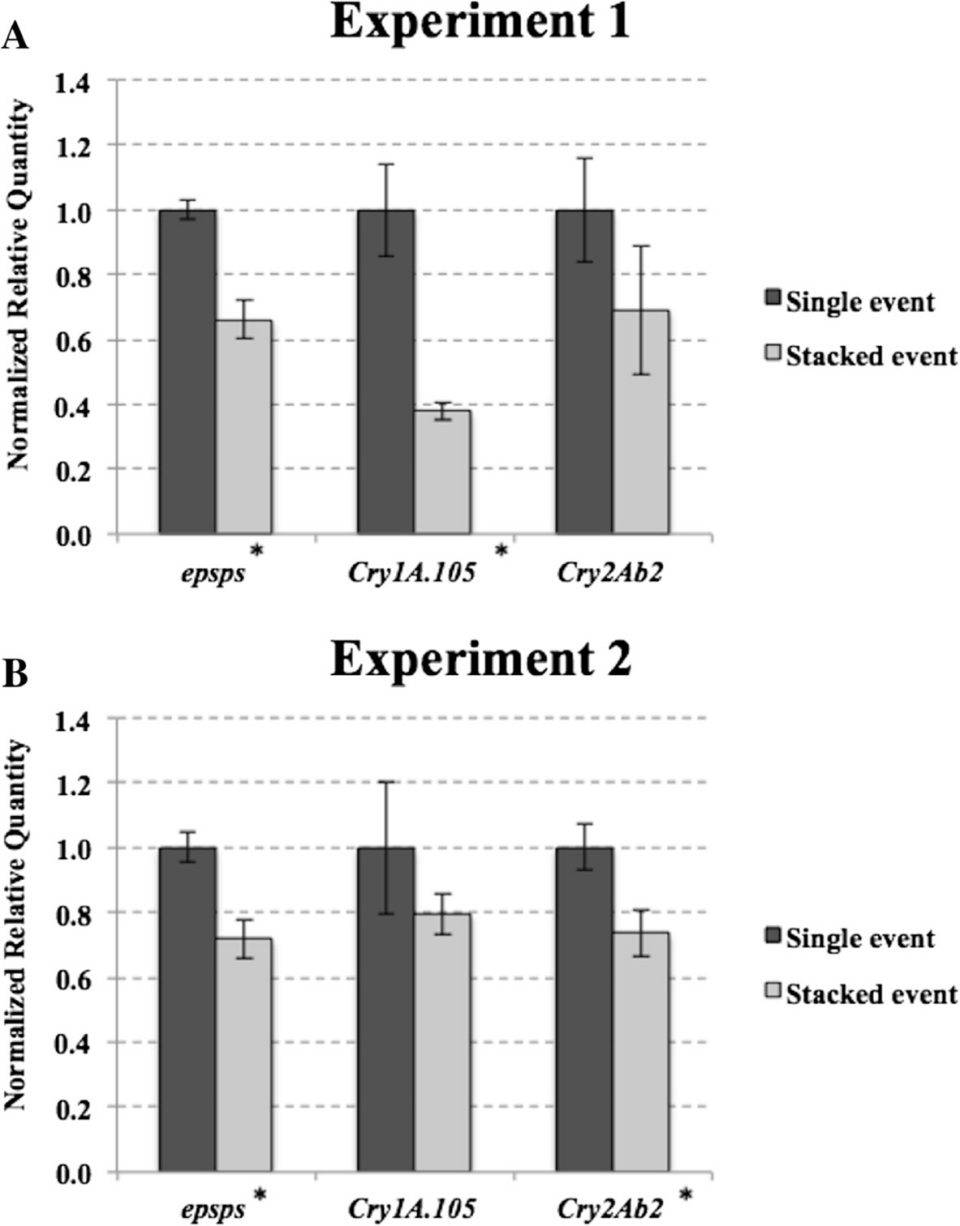
**Transgene transcripts normalized relative expression levels measured by delta-delta Cq method and Pffafl [** 19] **correction equation.** The *epsps*, *cry1A.105* and *cry2Ab2* transgenes were quantified from stacked versus single transgenic maize events grown under controlled conditions at V3 stage. Experiment 1 **(A)** and under the same conditions in Experiment 2 **(B)**. Samples are means of three pools, each derived from five different plants. ‘RR’ samples are transgenic maize seedlings from MON-ØØ6Ø3-6 event, ‘Bt’ samples are from MON-89Ø34-3 event, and ‘RRxBt’ samples are transgenic maize seedlings from MON-89Ø34-3 x MON-ØØ6Ø3-6 event. Bars indicate standard deviation and statistically significant values (*P* < 0.05) are represented by ‘*’.

A clear reduction of transcript levels for all three transgenes was observed in stacked compared to single events GM maize plants. Figure 1 shows normalized relative quantities for *epsps*, *cry1A.105* and *cry2Ab2* transcripts in both single and stacked events from experiment 1 (Figure 1A) and experiment 2 (Figure 1B). Performing experiment 2 under the same conditions reproduced the results of experiment 1. In fact, statistically significance was observed for *epsps* transcript in both experiments. Whereas experiment 1 had *cry1A.105* transcript and experiment 2 had *cry2Ab2* with statistically significant reduction, most probably due to biological variability observed by SD bars.

In the case of *epsps* transcripts, the average reduction in transgene accumulation was approximately 31%. Transcripts from *cry1A.105* showed reduction of transgene accumulation at an average of 41%, whereas *cry2Ab2* transcripts demonstrated a 29% reduction.

There is considerable variation in the expression of transgenes in individual transformants, which is not due to differences in copy number [30]. Nonetheless, the number of transgenes present in one genome can involve transgene/transgene interactions that might occur when homologous DNA sequences (e.g. expression controlling elements) are brought together [31]. Homology-dependent gene silencing has been revealed in several organisms as a result of the introduction of transgenes [32-36]. Gene silencing as a consequence of sequence duplications is particularly prevalent among plant species. The introduction of transgenes in plants produces at least two different homology-dependent gene-silencing phenomena: post-transcriptional gene silencing (PTGS) and transcriptional gene silencing (TGS) [37].

Typically, one transfer DNA (abbreviated T-DNA) exerts a dominant epigenetic silencing effect on another transgene on a second (unlinked) trans-acting coding T-DNA sequence. Silencing is often correlated with hyper-methylation of the silenced gene, which can persist after removal of the silencing insert. The results reported by Daxinger et al. [38] imply that gene silencing mediated by 35S promoter homology between transgenes and T-DNAs used for insertional mutagenesis is a common problem and occurs in tagged lines from different collections.

Homologous P35S promoters control the *epsps* and *cry1A.105* transgenes present in the stacked line used in this study. Whether silencing of 35S promoter in stacked events might be mediated by TGS or PTGS or other processes is not yet clear and requires further investigation.

Reduced transgene expression might also be related to the high energetic demand of the cell. In this regard, increasing evidences support the idea that constitutive promoters involve a high energetic cost and yields a penalty in transgenic plants [39-42]. In fact, results from research on salt tolerance suggest that the greater Na^+^ exclusion ability of the homozygous transgenic line over-expressing *HAL1* induces a greater use of organic solutes for osmotic balance, which seems to have an energy cost and hence a growth penalty that reverts negatively on fruit yield [42].

Nonetheless, changes in transgene expression levels in stacked events might affect their safety and utility. However, there is not enough data on the correlation between mRNA accumulation and transgenic protein levels. Therefore, further studies should be performed in order to investigate if reduced accumulation of transgene transcripts corresponds to reduced levels of Bt toxin production and the biological meaning behind these different levels of protein expression. One of the few examples of such investigation is the recent work of Koul et al. [43] on transgenic tomato line expressing modified *cry1Ab*, which showed correlation between transgene transcripts and protein levels in different plants. But while the bioassay results reflected a concentration-dependent response in the insect pest *Spodoptera litura*, the results on *Helicoverpa armigera* showed 100% mortality under different mRNA/protein concentrations [43]. The latter results give insights into possible uncorrelated biological relevance and protein levels for some target species.

Field-evolved resistance to Bt toxins in GM crops was first reported in 2006 for *S. frugiperda* in Puerto Rico [44]. Many other reported cases of field-resistance were confirmed as well [45]. The causes of such resistance were mainly related to the lack of compliance of growers that may not strictly adhere to the requirements for planting refuge areas with non-GM varieties [46,47]. Secondly, toxin doses might have been too low or variable to consistently kill heterozygous resistant insects [44,46,48,49]. Seasonal and spatial variation of Cry toxin content in GM cotton has been frequently linked to plant characteristics and environmental conditions [50]. In Bt maize, concentrations of Cry toxins have been shown to decline as the growing season progresses, but seasonal changes in toxin concentration are variable among toxins and cultivars [51]. The reasons for the seasonal reduction in Cry protein concentration remain unclear, but it could be related to mRNA instability, declining promoter activity, reduced nitrogen metabolism, lower overall protein production, and toxin interactions [52,53].

On the other hand, pyramiding two or more *cry* transgenes is expected to be more effective than single Cry toxins alone. It can reduce heritability of resistance and, thus, delay resistance [54]. But, declines in the concentration of one toxin in a pyramid could also invalidate the fundamental assumption of the pyramid strategy (i.e., the killing of insects resistant to one toxin by another toxin), and thus accelerate evolution of resistance [55]. Downes et al. [56] have provided a five-year data set showing a significant exponential increase in the frequency of alleles conferring Cry2Ab resistance in Australian field populations of *H. punctigera* since the adoption of a second generation, two-toxin Bt cotton.

Moreover, in cases where the expression level of an introduced/modified trait in a GM stacked event falls outside the range of what was determined in the parental line, a re-evaluation of the environmental aspects might be necessary, where considered relevant [3].

Monsanto submitted an approval application to the *Comissão Técnica Nacional de Biossegurança* (CTNBio, Brazil) for the stacked GM event employed in the present study. The document presented results of protein quantification for both stacked and single events, grown under farm conditions in three locations in Brazil [57]. The results show discrepancies for Cry and EPSPS protein levels determined by ELISA assay, in stacked versus single events. Leaves of single event plants (MON-89Ø34-3) had an average of 51, 24 and 24 ug.g^−1^ (fresh weight) for the three locations compared to 33, 26 and 38 ug.g^−1^ (fresh weight) of Cry2Ab2 protein in the stacked event plants (MON-89Ø34-3 x MON-ØØ6Ø3-6). Large variation (standard deviation values up to 19) and small sampling size (*N* = 4) must be taken into account and likely explain lack of statistical significance. To the best of our knowledge we are now presenting the first robust report on reduced levels of transgenic transcripts in commercial stacked GM varieties.

There is a lack of published data on transgene product expression levels in stacked versus single transgene GM crops in the scientific literature. Although data on expression levels for stacked GM events are required for approval according to EU regulations (N° 503/2013), these are rarely disclosed or they are considered insufficient [58-60].

### Proteomic profile of stacked RRxBt transgenic maize

The mean total protein content was 1.43 ± 0.6 mg.g^−1^ (fresh weight) of leaf material. No statistically significant difference was found between replicates and treatments. The genotype comparisons showed difference in the one-way ANOVA, followed by Tukey (*P* < 0.05). Conventional, landrace and Bt samples had higher amounts of total proteins content. Bt samples did not differ from RRxBt samples, which had higher amounts of total protein content compared to RR (Tukey HSD =0.76). The difference in the amount of extracted protein between plant genotypes did not affect the total number of spots resolved in the gel once sample loads were normalized to 80 ug per gel. The average number of spots detected (1123) on the 2D-DIGE gels showed similar patterns and they were considered well resolved for 24 cm fluorescent gel. No statistically significant differences (*P* < 0.05) were found between plant genotypes for number of spots detected.

In two dimensional gel electrophoresis, the lack of reproducibility between gels leads to significant system variability making it difficult to distinguish between technical variation and induced biological change. On the other hand, the methodological approach used in the present work, called 2D-DIGE, provides a platform for controlling variation due to sample preparation, protein separation and difference detection by fluorescent labeling and the co-migration of treatment and control samples in the same gel [61-63]. Nonetheless, each 2D-DIGE run consisted of three samples, two of which were randomly selected from all plant variety samples and one being an internal standard used in all runs for normalization purposes.

### Principal Component Analysis (PCA)

**Figure 2:**
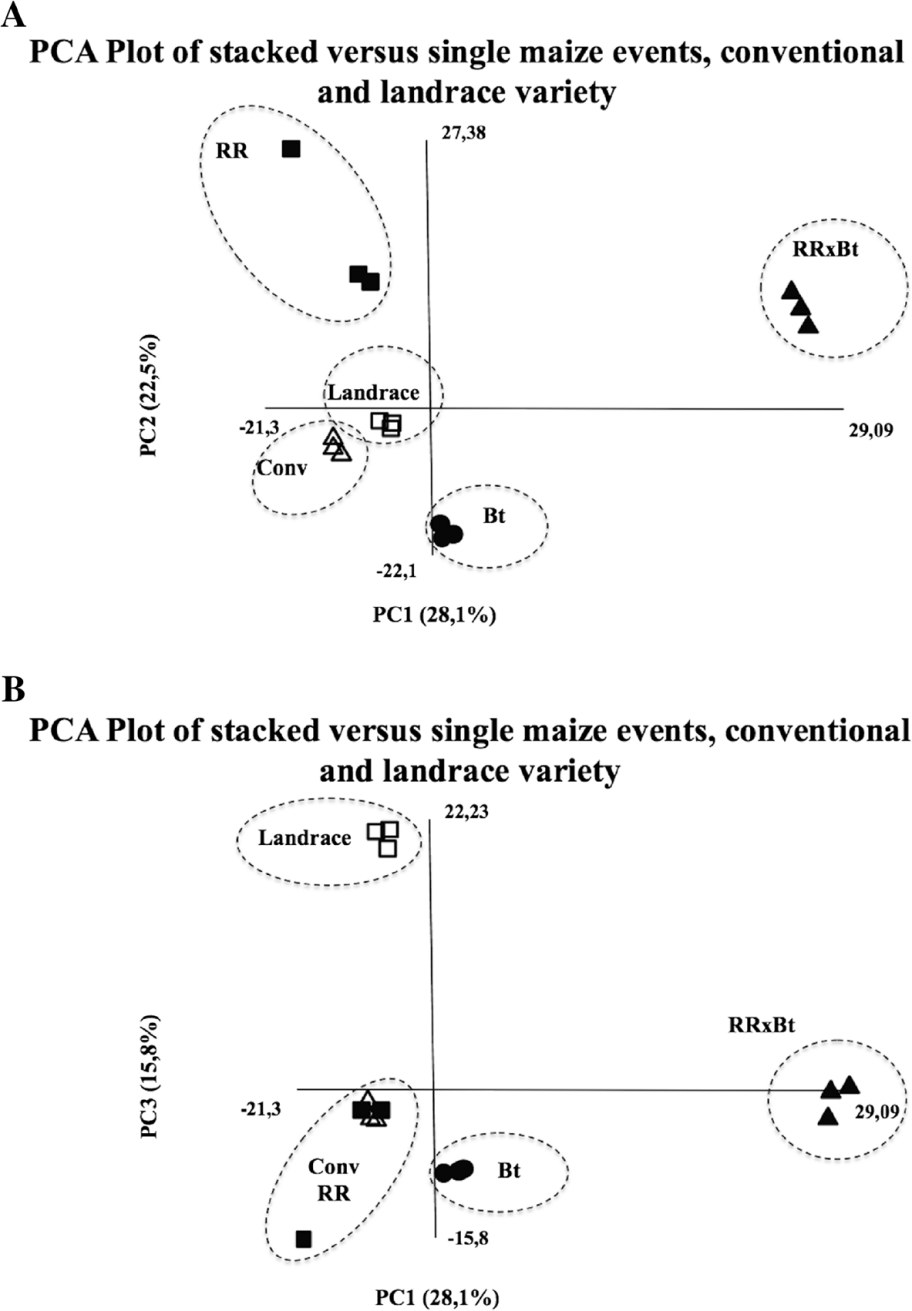
**PCA score plots of proteome data of genetically modified stacked and single events, non-genetically modified near-isogenic variety, and landrace maize variety.** Proteome data was obtained by 2D-DIGE analysis from leaf material of maize plants grown under controlled conditions. PC1 and PC2 **(A)** and PC1 and PC3 **(B)** show the results of ‘RR’ samples (transgenic maize seedlings from MON-ØØ6Ø3-6 event, filled squares), ‘Bt’ samples (MON-89Ø34-3 event, filled circles), ‘RRxBt’ samples (transgenic maize seedlings from MON-89Ø34-3 x MON-ØØ6Ø3-6 event, filled triangles), ‘CONV’ samples (conventional non-transgenic near isogenic maize variety, blank triangles), and ‘landrace’ (Pixurum 5 landrace variety, blank squares).

PCA was used to demonstrate similarities in protein quantity between different gels and to gain insight into possible proteome x transgene interactions in the dataset. In the analysis of the PCA, the first four eigenvalues corresponded to approximately 80% of accumulated contribution. All fifteen samples were represented 2-dimensionally using their PC1, PC2 and PC3 scores (in two separated plots), revealing groups of samples based on around 66% of all variability (Figure 2A and B). This analysis showed a complete separation in the first plot (PC1 × PC2) between the transgenic events containing insecticidal Cry proteins and other maize varieties that do not express those (the conventional, the landrace and the RR transgenic event), which explained 28.1% of the total variation (F1 values below −21.3 and above +29.9, respectively). PC2 explained 22.5% of the variation and showed a separation of plant genotypes containing RR transgene.

The results from our previous investigation, using another Bt event (MON-ØØ81Ø-6) grown under two different agroecosystems, showed that the environment was the major source of influence to the maize proteome and accounted for 20% of the total variation. However, the different genotypes (Bt and comparable conventional) accounted for the second major source of variability, about 9% [25].

Barros et al. [64] used the same RR transgenic event utilized in the present study and a different Bt event (MON-ØØ81Ø-6) in the same genetic background and found an interesting proteomic pattern that accounted for 31% of the total variation in their dataset. RR maize samples were grouped separately from Bt and conventional samples grown at field conditions. This pattern was also observed in their microarray and gas chromatographic/mass spectrometric metabolite profile analysis. Even when the environment or the plant genetic background accounts for the majority of the quantitative data variation, transgenic and their conventional near-isogenic varieties are frequently observed in separated groups by PCA [65].

In our second plot (PC1 × PC3) another clear separation was observed for landrace samples, thus explaining 15.6% of the variation in the full dataset (Figure 2B). Unexpectedly, the landrace variety did not account for the majority of the variation in the dataset. There was no variation between biological replicates within each plant variety, but pool 2 from RR samples seems to deviate from other replicates.

Although 66.2% of the variation might represent the majority of the total variation, care must be taken when interpreting these results because other sources of variation might be present in subsequent factors.

A landrace variety was included in this study in order to consider the extent of proteomic variation related to different maize genetic backgrounds, as well as to possibly disclose differences in GM lines that might fit within the variation observed in non-modified materials. It should be emphasized that the use of non-GM varieties that are genetically distant from the GM event under investigation is not a requirement of international guidelines addressing the issue for comparative assessments of the environmental and health risk analysis of GM plants [7]. Thus, the presence of a biological relevant difference unique to the GMO being evaluated does not depend on the overall variation observed in particular environment × gene scenarios or breeding conditions [11].

A landrace variety was also included in a comparative analysis of potato tuber proteomes of GM potato varieties by Lehesranta et al. [66]. These authors found extensive genotypic variation when analyzing around 25 GM, non-GM and landrace varieties. Most of the proteins detected exhibited significant quantitative and qualitative differences between one or more variety and landraces. Unfortunately, these authors did not plot all the varieties in the same PCA.

Taken together, these results demonstrated the relevance of detecting major sources of variation in the experimental dataset. Thus, for benchmarking and comparative analysis approaches, the deployment of broader scale, less biased analytical approaches for GM safety assessment should also embrace the issues of sources and extents of variation [67].

It has already been demonstrated that major changes in the proteomic profile of GM crops are driven by genotypic, environmental (geographical and seasonal) and crop management influences (and combinations thereof) rather than by insertional transgenetic engineering. However, it has also been observed that the genetic engineering does have an influence in the modulation of certain proteins and pathways thereby [68]. Furthermore, off-target effects of GM crops have also been evidenced at different levels and some do not directly correspond to the levels of transgenic protein expression [69]. In some cases, beneficial effects of the transgene might be influenced by pleiotropic effects derived from the use of strong promoters and new proteins [70-72].

### Mass spectral identification of differentially expressed proteins

Comparison of stacked and single GM varieties, in the same genetic background, and non-GM varieties (the near-isogenic conventional counterpart and a landrace) revealed a total of 22 different proteins that were either present, absent, up- or down-regulated in one of the hybrids, at a statistically significant level (*P* < 0.05) (Table 2). Proteins that were not detected in this study might not be expressed or fall below the detection limit of approximately 1 ng, and were then considered absent in the sample.

**Table 2:** **Differentially expressed proteins in stacked transgenic maize variety versus controls (single event transgenic maize variety with the same genetic background) and non-genetically modified counterpart and a landrace by 2D-DIGE analysis**

**Match ID**	**Genebank ID**	**Protein name**	**Mascot score**	**Sequence coverage (%)**	**Peptides**	**Theor. mass (kDa)**	**Theor. pI (pH)**	**Exp. mass (kDa)**	**Exp. pI (pH)**	**Fold change (ANOVA** ***P*** **< 0.05)**	**Biological process (KEGG Orthology)**
55	gi|11467199	ATP synthase CF1 beta subunit [*Zea mays*]	2248	72	62	54	5.31	56	5.80	Conv, RR, RRxBt > Bt > Land	Metabolism (energy metabolism)
155	gi|413948212	hypothetical protein ZEAMMB73_661450 [*Zea mays*]	723	44	21	46	5.62	44	5.96	Land > Conv, RR, Bt, RRxBt	Metabolism (energy metabolism)
156	gi|413939324	glutamate-oxaloacetate transaminase2 [*Zea mays*]	1201	61	43	50	8.43	44	6.12	Land > Bt > Conv, RR, RRxBt	Metabolism (carbohydrate metabolism; biosynthesis of amino acids)
231	gi|195622374	fructose-bisphosphate aldolase [*Zea mays*]	798	40	19	40	5.39	37	5.50	Land > Conv, RR, Bt, RRxBt	Metabolism (carbohydrate metabolism; biosynthesis of amino acids)
406	gi|414591286	APx2-cytosolic ascorbate peroxidase [*Zea mays*]	1036	59	20	31	5.77	27	5.78	Conv, RR, Bt, RRxBt > Land	Metabolism (carbohydrate metabolism; biosynthesis of amino acids)
426	gi|226504576	APx1 - cytosolic ascorbate peroxidase [*Zea mays*]	772	54	18	27	5.65	26	5.74	Bt > Conv > RRxBt > RR > Land	Metabolism (carbohydrate metabolism; biosynthesis of amino acids)
171	gi|414586172	3-isopropylmalate dehydrogenase [*Zea mays*]	1042	44	23	43	5.62	42	5.18	Conv > Bt > Land> RR > RRxBt	Metabolism (biosynthesis of amino acids)
175	gi|195645514	acyl-desaturase [*Zea mays*]	441	24	8	45	6.61	42	6.15	Bt > Conv, RR, RRxBt > Land	Metabolism (fatty acid metabolism)
177	gi|308081433	coproporphyrinogen III oxidase [*Zea mays*]	321	24	9	47	7.23	42	6.12	Conv, Bt > RR, RRxBt > Land	Metabolism (cofactors and vitamins metabolism)
762	gi|226499080	dihydroflavonol-4-reductase [*Zea mays*]	534	36	14	35	5.43	33	5.83	RR > Conv, Bt, RRxBt > Land	Metabolism (biosynthesis of other secondary metabolites)
64	gi|226492645	vacuolar ATP synthase subunit B [*Zea mays*]	711	45	17	54	5.07	54	5.19	Bt, RRxBt > Conv, Land, RR	Metabolism (energy metabolism); Cellular Processes (transport and catabolism; phagosome)
105	gi|162458207	enolase 1 [*Zea mays*]	1604	67	29	48	5.20	49	5.60	RR > Bt > Conv > RRxBt > Land	Metabolism (carbohydrate metabolism; biosynthesis of amino acids); Genetic Information Processing (Folding, sorting and degradation); Environmental Information Processing (signal transduction)
437	gi|413951084	hypothetical protein ZEAMMB73_536198 [*Zea mays*]	416	32	9	28	5.14	26	5.39	Land > Conv > Bt > RR > RRxBt	Metabolism (metabolism of cofactors and vitamins); Genetic Information Processing (transfer RNA biogenesis)
714	gi|195619804	enolase [*Zea mays*]	663	40	16	48	5.59	56	6.05	Land > Conv, Bt, RRxBt > RR	Metabolism (carbohydrate metabolism; biosynthesis of amino acids); Genetic Information Processing (Folding, sorting and degradation); Environmental Information Processing (signal transduction)
137	gi|226505740	DIMBOA UDP-glucosyltransferase BX9 [*Zea mays*]	1197	49	23	50	5.15	45	5.43	RR > Conv, RRxBt > Bt > Land	Metabolism (biosynthesis of other secondary metabolites); Genetic Information Processing (folding, sorting and degradation)
415	gi|414591366	6-phosphogluconolactonase isoform 1 [*Zea mays*]	333	28	7	35	7.71	26	5.08	Conv, RR, Bt, RRxBt > Land	Metabolism (carbohydrate metabolism; biosynthesis of amino acids)
421	gi|195611274	14-3-3-like protein [*Zea mays*]	858	67	23	29	4.82	26	4.93	Bt, RRxBt > Conv, Land, RR	Environmental Information Processing (signal transduction); Cellular Processes (cell growth and death); Exosome (exosomal protein)
572	gi|226504688	uncharacterized protein LOC100272933 precursor [*Zea mays*]	202	13	9	22	6.02	19	6.62	Bt, RRxBt only	Metabolism (carbohydrate metabolism)
345	gi|195619262	gibberellin receptor GID1L2 [*Zea mays*]	244	20	5	33	4.93	31	5.06	Bt, RRxBt only	Environmental Information Processing (signal transduction)
545	gi|195626524	2-cys peroxiredoxin BAS1 [*Zea mays*]	160	23	4	28	5.81	21	4.56	Bt, RRxBt only	Cellular Processes (transport and catabolism)
38	gi|226493235	LOC100281701 [*Zea mays*]	1110	41	17	61	5.20	59	5.15	RR only	Genetic Information Processing (folding, sorting and degradation)
750	gi|226530174	ankyrin repeat domain-containing protein 2 [*Zea mays*]	619	57	16	38	4.57	36	4.66	RR only	Genetic Information Processing (folding, sorting and degradation)

Proteins were considered differentially modulated at statistical significant difference in normalized volume in stacked vs. single GM events and control samples at ANOVA *P* < 0.05. Proteins were classified in functional categories based on the ExPASy, KEGG Orthology databases and on careful literature evaluation. The Table reports spot number (Match ID), accession number and protein name, together with Mascot score, sequence coverage, number of matched peptides, theoretical and experimental molecular weight (MW), isoelectric point (pI) and fold change. Abbreviations for each plant variety are provided within ‘Methods’ section.

**Figure 3:**
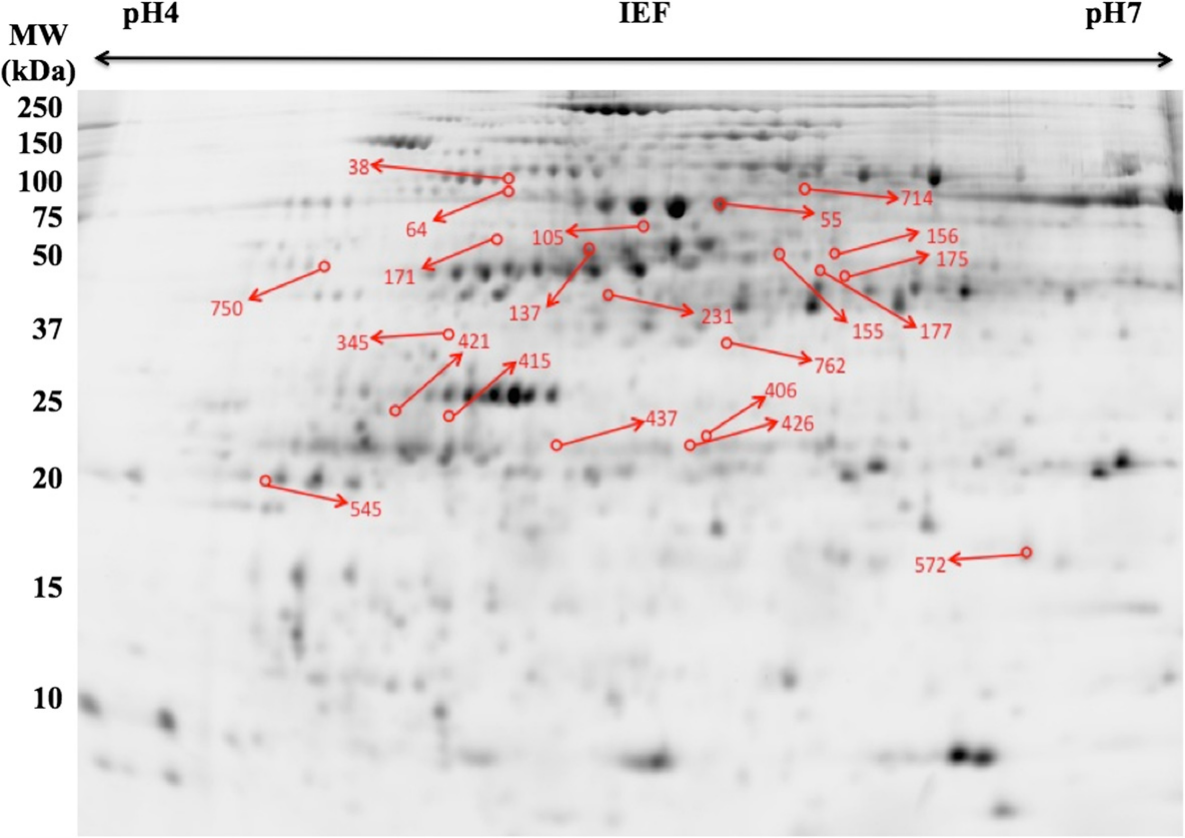
**Representative 24 cm two-dimensional gel electrophoresis (2D-DIGE) image of the proteome of genetically modified maize plant leaves AG8025 hybrid varieties MON-89Ø34-3 and MON-ØØ6Ø3-6 single events, and MON-89Ø34-3 x MON-ØØ6Ø3-6 stacked event, and non-modified maize (conventional counterpart AG8025 hybrid variety and landrace Pixurum 5 variety) grown under controlled conditions.** Two random replicate samples were run together with an internal standard sample, each labeled with a different fluorescence. Individualgel images were obtained and plotted together using ImageQuant TL software from GE healthcare. Linear isoelectric focusing pH 4–7 for the first dimension and 12% SDS–PAGE gels in the second dimension were used. Molecular mass standard range from 250 to 10 kDa are given on the left side. Red arrows point to differentially expressed protein spots selected for mass spectrometry identification. ID of identified proteins from Table 2 are indicated in red numbers.

All 22 proteins were identified with Mascot scores value greater than 202 using Quadrupole Time-of-Flight (Q-TOF) tandem mass spectrometry analysis (MS/MS) (*P* < 0.05). These proteins were all identified in *Zea mays* species. Table 2 presents the MS/MS parameters and protein identification characteristics for this experiment, while Figure 3 show their location in a representative gel. It was found that 17 proteins differed in their expression levels between genotypes and 5 were found to be present only in one or two specific genotypes. Normalized quantitative values for each of these proteins and statistic analysis are present in Table 3.

**Table 3:** **Relative protein expression levels analysis of differentially modulated (** ***P*** **< 0.05) proteins measured by 2D-DIGE analysis**

**Match**	**Conventional**	**Landrace**	**RR**	**Bt**	**RRxBt**
55	0.713 a	0.511 b	0.804 a	0.621 ab	0.731 a
64	0.934 b	0.920 b	0.831 b	1.161 a	1.097 a
105	0.865 abc	0.647 c	0.994 a	0.948 ab	0.704 bc
137	0.934 ab	0.646 c	1.174 a	0.816 bc	0.974 ab
155	0.696 b	0.939 a	0.782 b	0.775 b	0.694 b
156	0.709 b	0.949 a	0.778 b	0.837 ab	0.725 b
171	1.375 a	1.181 abc	0.954 bc	1.272 ab	0.921 c
175	0.928 ab	0.659 b	0.807 ab	0.981 a	0.926 ab
177	1.035 a	0.555 b	0.857 ab	0.898 a	0.815 ab
231	0.891 b	1.090 a	0.793 b	0.860 b	0.905 b
406	1.157 a	0.696 b	1.169 a	1.074 a	1.027 a
415	0.862 a	0.330 b	1.192 a	0.947 a	1.032 a
421	0.739 b	0.652 b	0.750 b	0.997 a	0.847 ab
426	0.993 ab	0.780 c	0.851 bc	1.077 a	0.902 abc
437	1.055 ab	1.077 a	0.887 bc	0.977 abc	0.812 c
714	0.910 ab	0.954 a	0.650 b	0.880 ab	0.765 ab
762	0.880 ab	0.467 b	1.228 a	0.850 ab	0.914 ab
345	-	-	-	1.119a	0.676b
545	-	-	-	0.709b	0.806a
572	-	-	-	0.945a	0.688b
38	-	-	0.920	-	-
750	-	-	1.248	-	-

Modulations are reported as normalized spot volume in stacked vs. single GM event plants and control samples. Tukey Test was applied at *P* < 0.05 for means separation and statistical significance. The different letters represents statistically significant mean values. For the last 5 spots (345, 545, 572, 38 and 750) missed values in protein abundance is not reported because these proteins were not detected in these respective plant varieties. Protein identities are provided in Table 2 according to their Match ID number.

Functional classification of the identified proteins, carried out in accordance with the KEGG Orthology system database, showed that they were assigned to one out of these four main ortholog groups: (a) Metabolism (Energy, Carbohydrate and biosynthesis of amino acid, Fatty acid, Cofactors and vitamins, Secondary metabolites), (b) Cellular Processes (Transport and catabolism, Cell growth and death), (c) Genetic Information Processing (Folding, sorting and degradation, Transfer RNA biogenesis), and (d) Environmental Information Processing (Signal transduction). The ‘Metabolism’ group constituted the major category for all proteomes (77% of all identified proteins), although represented by different proteins.

We have performed an enrichment analysis in order to rank associations between our set of identified proteins representing metabolic pathways with a respective statistical probability (Table 4). The results show that only seven proteins were assigned to statistically significant pathways. These pathways can be grouped into two main categories: the energy/carbohydrate metabolism (glycolysis, gluconeogenesis, tricarboxylic acid cycle – TCA cycle, glucose and xylose degradation, and L-ascorbate degradation) and the detoxification metabolism (ascorbate glutathione cycle). These will be discussed separately in the following sections.

**Table 4:** **BioCyc database collection enrichment analysis for the differentially expressed proteins in stacked vs. single GM event maize plants and control samples**

**Pathway term**	***P*** **-values**	**Proteins assigned to the pathway**
Glycolysis	7.538e-4	fructose-bisphosphate aldolase; 14-3-3-like protein; enolase; enolase 1.
Gluconeogenesis	8.781e-4	fructose-bisphosphate aldolase; 14-3-3-like protein; enolase; enolase 1.
Superpathway of cytosolic Glycolysis (plants), Pyruvate Dehydrogenase and TCA Cycle	0.006	fructose-bisphosphate aldolase; 14-3-3-like protein; enolase; enolase 1.
Superpathway of Anaerobic Sucrose Degradation	0.007	fructose-bisphosphate aldolase; 14-3-3-like protein; enolase; enolase 1.
Sucrose Degradation	0.011	fructose-bisphosphate aldolase; 14-3-3-like protein; enolase; enolase 1.
L-Ascorbate Degradation	0.003	APx1 - cytosolic ascorbate peroxidase; APx2-cytosolic ascorbate peroxidase.
Ascorbate Glutathione Cycle	0.004	APx1 - cytosolic ascorbate peroxidase; APx2-cytosolic ascorbate peroxidase.
Glucose and Xylose Degradation	0.006	6-phosphogluconolactonase isoform 1; enolase; enolase 1.

The identified pathways were searched against the maize (*Zea mays mays*) genome database at statistical level of *P* < 0.01.

Five exclusive proteins that belong to different protein families were identified through a detailed interpretation of all identified proteins. These are: cupin family (uncharacterized protein LOC100272933 precursor - Bt and RRxBt samples; carbohydrate metabolism), esterase and lipase family (gibberellin receptor GID1L2 - Bt and RRxBt samples; environmental information processing), peroxiredoxin family (2-cys peroxiredoxin BAS1 - Bt and RRxBt samples; transport and catabolism), chaperonin family (LOC100281701 - RR samples; genetic information processing), and ankyrin repeat family (ankyrin repeat domain-containing protein 2 - RR samples; genetic information processing).

Six proteins were differentially expressed in landrace only. These are ATP synthase CF1 beta subunit (Match ID 55), hypothetical protein ZEAMMB73_661450 (Match ID 155), glutamate-oxaloacetate transaminase2 (Match ID 156), fructose-bisphosphate aldolase (Match ID 231), APx2-cytosolic ascorbate peroxidase (Match ID 406) and 6-phosphogluconolactonase isoform 1 (Match ID 415).

Enolase proteins were also assigned to two other spots (Match ID 105 and 714), the latter was expressed at higher levels in single GM events. ATP synthase, which was identified in spots ID 55 and 64, was expressed at a higher level in the vacuole of mono-transgenic Bt maize. These proteins are considered to represent different protein isoforms resulting from posttranslational modifications that introduce changes of molecular weight (MW) and/or isoelectric point (pI).

### Proteins related to energetic homeostasis

The identity of proteins related to the energetic metabolism can be found in Table 2. They belong to the protein families of ATP synthases, NADH dehydrogenases, aminotransferases, fructose-bisphosphate aldolases, peroxidases, isopropylmalate dehydrogenases, enolases and the cupin family. Except for the cupin protein that was only detected in Bt and RRxBt samples, all proteins were present in all samples at different levels of expression.

The enrichment analysis provided insight into major pathways alteration; gluconeogenesis, glucose, xylose and L-ascorbate degradation are key processes for conversion of various carbon sources into nutrients and energy.

Enzymes that catalyze such chemical reactions were already observed in other comparative proteomic studies of transgenic versus non-transgenic crops. In fact, the energetic metabolism, including the carbohydrate metabolism, has been the most frequently observed protein category within comparative analysis of transgenic versus non-transgenic crops (see compilation at Table 3 from Agapito-Tenfen et al. [25]).

A detailed analysis of each protein separately shows interesting modulation patterns. Enolase enzymes that participate in the glycolysis pathway were differentially modulated in single versus stacked GM events (Match ID 105 and 714). For spot 105, RRxBt samples showed reduced expression levels compared to single GM events and the conventional variety, while spot 714 was less abundant in RR samples. Barros et al. [64] also found differential modulation of enzymes related to the glycolysis by analyzing gene expression mean levels (3 years) obtained by microarray profiling of maize grown in South Africa. The results demonstrated that glyceraldehyde 3-phosphate dehydrogenase was expressed at higher levels in Bt-transgenic plants than in non-transgenic and RR samples. Furthermore, Coll et al. [73] observed lower levels of triose-phosphate isomerase protein, also a glycolysis enzyme, in Bt-transgenic plants than in their non-transgenic counterpart. Indeed, the flux through of the glycolysis metabolic pathway can be regulated in several ways, i.e. through availability of substrate, concentration of enzymes responsible for rate-limiting steps, allosteric regulation of enzymes and covalent modification of enzymes (e.g. phosphorylation) [74]. Currently, the transcriptional control of plant glycolysis is poorly understood [75]. Studies on transgenic potato plants exhibiting enhanced sucrose cycling revealed a general upregulation of the glycolytic pathway, most probably mediated at the level of transcription [75].

Higher levels of sucrose and fructose were observed in Bt-transgenic maize plants than in RR transgenic maize and non-transgenic samples obtained by H-NMR-based metabolite fingerprinting [64].

Intensive nuclear functions, such as transgenic DNA transcription and transport of macromolecules across the nuclear envelope, require efficient energy supply. Yet, principles governing nuclear energetics and energy support for nucleus-cytoplasmic communication are still poorly understood [76,77]. Dzeja et al. [77] have suggested that ATP supplied by mitochondrial oxidative phosphorylation, not by glycolysis, supplies the energy demand of the nuclear compartment.

Higher expression levels of ATP synthase, an enzyme that participates in the oxidative phosphorylation pathway, were observed in Bt and RRxBt plants compared to Bt and conventional (Match ID 64). Regarding 3-isopropylmalate dehydrogenase (Match ID 171), which is related to the TCA cycle, it was differentially modulated in all GM events, whereas plants expressing the stacked event had lower levels compared to Bt single GM event, and RR samples had intermediate levels.

### Proteins related to other cellular metabolic pathways and processes

Proteins assigned to other pathways than those related to the energetic metabolism were grouped in this section. The enrichment analysis revealed an additional major metabolic pathway, i.e. the ascorbate-glutathione cycle, which is part of the detoxification metabolism in plants. Thus, ascorbic acid acts as a major redox buffer and as a cofactor for enzymes involved in regulating photosynthesis, hormone biosynthesis, and regenerating other antioxidants [78].

Other identified proteins are enzymes related to fatty acid, vitamin and secondary metabolite metabolism; transport and catabolism and cell growth and death; folding, sorting and degradation of nucleic acids; and signal transduction. Table 3 shows expression levels obtained by 2D-DIGE experimentation.

Coproporphyrinogen III oxidase and S-adenosyl methionine (SAM) (Match ID 177 and 437) are an important enzyme and co-factor, respectively, that act within the metabolism of vitamins in plants. They were modulated in similar manners in each maize variety, with higher expression in the conventional variety. The former enzyme plays an important role in the tetrapyrrole biosynthesis that is highly regulated, in part to avoid the accumulation of intermediates that can be photoactively oxidized, leading to the generation of highly reactive oxygen intermediates (ROI) and subsequent photodynamic damage [79]. SAM plays a critical role in the transfer of methyl groups to various biomolecules, including DNA, proteins and small-molecular secondary metabolites [80]. SAM also serves as a precursor of the plant hormone ethylene, implicated in the control of numerous developmental processes [81].

Two other proteins related to the synthesis of secondary metabolites were expressed at statistically different levels. These are Match ID 137 and 762.

It has been observed that both these enzymes are expressed at higher levels in all hybrid plants (GM and non-GM) than in the landrace samples. DIMBOA UDP-glucosyltransferase BX9 is an enzyme that participates in the synthesis of 2,4-Dihydroxy-7-methoxy-1,4-benzoxazine- 3-one (DIMBOA) compound that plays an important role in imparting resistance against disease and insect pests in gramineous plants [82] as well as herbicide tolerance [83]. DIMBOA decreases *in vivo* endoproteinase activity in the larval midgut of the European corn borer (*Ostrinia nubilalis*), limiting the availability of amino acids and reducing larval growth [84,85]. The protection against insect attack that DIMBOA confers to the plant is, however, restricted to early stages of plant development, because DIMBOA concentration decreases with plant age [86-88]. The other enzyme related to the metabolism of secondary metabolites follows exactly the same trend in expression. Dihydroflavonol-4-reductase catalyzes a key step late in the biosynthesis of anthocyanins, condensed tannins (proanthocyanidins), and other flavonoids, important for plant survival, including defense against herbivores [89].

Two enzymes related to genetic information processing were observed in RR samples only. Match ID 750 was identified to contain an ankyrin repeat domain. The ankyrin repeats are degenerate 33-amino acid repeats found in numerous proteins, and serve as domains for protein-protein interactions [90]. By using antisense technique, Yan et al. [91] were able to reduce the expression levels of an ankyrin repeat-containing protein, which resulted in small necrotic areas in leaves accompanied by higher production of H_2_O_2_. These results were found to be similar to the hypersensitive response to pathogen infection in plant disease resistance [91]. Although we were not able to identify an annotated protein to Match ID 38, blast results show that this protein belong to the chaperonin protein family. Chaperones are proteins that assist the non-covalent folding or unfolding and the assembly or disassembly of other macromolecular structures. Therefore, cells require a chaperone function to prevent and/or to reverse incorrect interactions that might occur when potentially interactive surfaces of macromolecules are exposed to the crowded intracellular environment [92]. A large fraction of newly synthesized proteins require assistance by molecular chaperones to reach their folded states efficiently and on a biologically relevant timescale [93].

Another relevant class of enzymes is linked to plant perception and response to environmental conditions (environmental information processing). An important protein of this category is gibberellin receptor GID1L2 (Match ID 345). Gibberellins (GAs) are hormones that are essential for many developmental processes in plants, including seed germination, stem elongation, leaf expansion, trichome development, pollen maturation and the induction of flowering [94]. This protein was only detected in Bt-transgenic plant samples and RRxBt samples).

### Contributions to the risk assessment of stacked transgenic crop events

Recent discussions about potential risks of stacked events, as well as the opinion of the European Food Safety Authority (EFSA) on those issues, have highlighted the lack of consensus with regard to whether such GMOs should be subject to specific assessments [59]. Similar debates have taken place in the Brazilian CTNBio, while approving stacked GM events under a simplified risk assessment procedure provided by Normative Resolution n^o^ 8 from 2009 [4].

As for the above-mentioned regulatory bodies, both considered the need for a comparative evaluation of transgene expression levels in stacked GM event versus parental events (single events that have been crossed to produce the stacked event), and the need to consider any potential interaction of combined GM traits in the stacked events.

It is clear, for reasons discussed previously in this paper, that expression levels of stacked GM events are of major concern. On the other hand, testing potential interactions of stacked transgenic proteins, and of genetic elements involved in its expression, is an obscure issue and simple compositional analysis and/or evaluation of agronomic characteristics might not make contributions to further clarification.

Molecular profiling at the hazard identification step can fill the biosafety gap emerging from the development of new types of GMOs that have particular assessment challenges [11].

Over the past few years a number of published studies have used general “omics” technologies to elucidate possible unintended effects of the plant transformation event and transgene expression [12,95-97]. These studies have mainly compared single events with their non-transgenic near-isogenic conventional counterpart.

So far, no other study has compared differentially expressed proteins in stacked GM maize events and their parental single event hybrids and non-transgenic varieties. Hence, there is a lack of data of a kind that might be important in order to reliably assess the safety of stacked GM events.

## Conclusions

In conclusion, our results showed that stacked GM genotypes were clustered together and distant from other genotypes analyzed by PCA. In addition, we obtained evidence of possible synergistic and antagonistic interactions following transgene stacking into the GM maize genome by conventional breeding. This conclusion is based on the demonstration of twenty-two proteins that were statistically differentially modulated. These proteins were mainly assigned to the energy/carbohydrate metabolism (77% of all identified proteins). Many of these proteins have also been detected in other studies. Each of those was performed with a different plant hybrid genotype, expressing the same transgene cassette, but grown under distinct environmental conditions. Moreover, transgenic transcript accumulation levels demonstrated a significant reduction of about 34% when compared to parental single event varieties. Such observations indicate that the genome changes in stacked GM maize may influence the overall gene expression in ways that may have relevance for safety assessments. Some of the identified protein modulations fell outside the range of natural variability observed in a commonly used landrace. This is the first report on comparative proteomic analysis of stacked versus single event transgenic crops. However, the detection of changed protein profiles does not present a safety issue *per se*, but calls for further studies that address the biological relevance and possible safety implications of such changes.

## Additional file

Additional file 1:
**Description of candidate reference genes and transgenes, their primer sequences, gene product and Genebank accession number.**
