# Supplementary material for: Effect of stacking insecticidal cry and herbicide tolerance epsps transgenes on transgenic maize proteome
Source: BMC Plant Biol. 2014 Dec 10;14:346. doi: 10.1186/s12870-014-0346-8 (PMC4273480; doi:10.1186/s12870-014-0346-8)
Supplement: Additional file 1: — Description of candidate reference genes and transgenes, their primer sequences, gene product and Genebank accession number. [file 12870_2014_346_MOESM1_ESM.docx]

Additional file 1. Description of candidate reference genes and transgenes, their primer sequences, gene product and Genebank accession number.

| **Primer name** | **Gene product** | **Genbank Accession no.** | **Primer sequence** |
| --- | --- | --- | --- |
| MEP | Membrane protein PB1A10.07c | GRMZM2G018103 T01 | F - GTACTCGGCAATGCTCTTGA |
|  |  |  | P - AACTTCGGTTGGTGAGAGCGGAAA |
|  |  |  | R - CAATCCTGACCCAGACAGATG |
| LUG | Leunig | GRMZM2G425377 T01 | F - GGGACATAAGGGAGAAGAACAC |
|  |  |  | P - TTCCCTGTAGCACTGGATGATGCC |
|  |  |  | R - TCATGGCTTACTGAGGCAAC |
| CUL | Cullin | GRMZM2G166694 T04 | F - CGACAAGGACAACGCCAATA |
|  |  |  | P - ACCTTGCCTGATTGGTGGTTAGTGA |
|  |  |  | R - TCCCAGTGGTATCGCATAGT |
| FPGS | Folylpolyglutamate synthase | GRMZM2G393334 T01 | F - CTTTCCAGGTGCTGGTTACT |
|  |  |  | P - TCAAGAAGTGATACGCCGCTCGAA |
|  |  |  | R - TCATAGTCCAGTTCCAGTTTGG |
| UBPC | Ubiquitin carrier protein | GRMZM2G102471 T01 | F - ACAGTGGAGTCCTGCTTTAAC |
|  |  |  | P - TCAATCTGCTCACTGCTCACGGAC |
|  |  |  | R - GAGCAATCTCAGGGACAAGAG |
| EPSPS | CP4-epsps protein | Company document only | F - TACGATTTCGACAGCACCTTC |
|  |  |  | P - TTGAACCCGCTGCGCGAAATG |
|  |  |  | R - GTCACCGTCTTCCGATTTCA |
| cry1A.105 | cry1A.105 protein | FV532179 | F - GACGTGGAGGAACAGAACAA |
|  |  |  | P - TTGTGCCTGAGTGGGAAGCTGAA |
|  |  |  | R - CCTCTACCTGGACAGACTCTAA |
| cry2Ab2 | cry2Ab2 protein | FV532179 | F - GCGACTACCTGAAGAACTACAC |
|  |  |  | P - CAACACCTACCAGTCGGCCTTCAA |
|  |  |  | R - TGTCGTGAAGCCTCGTATTG |
